# *IL4* gene polymorphism and previous malaria experiences manipulate anti-*Plasmodium falciparum *antibody isotype profiles in complicated and uncomplicated malaria

**DOI:** 10.1186/1475-2875-8-286

**Published:** 2009-12-10

**Authors:** Piyatida Tangteerawatana, Hedvig Perlmann, Masashi Hayano, Thareerat Kalambaheti, Marita Troye-Blomberg, Srisin Khusmith

**Affiliations:** 1 Department of Microbiology and Immunology, Faculty of Tropical Medicine, Mahidol University, 420/6 Rajvithi Road, Bangkok 10400, Thailand; 2 Department of Microbiology, Faculty of Medicine, Srinakharinwirot University, Bangkok, Thailand; 3 Department of Immunology, Wenner-Gren Institute, Stockholm University, Stockholm, Sweden

## Abstract

**Background:**

The *IL4*-590 gene polymorphism has been shown to be associated with elevated levels of anti-*Plasmodium falciparum *IgG antibodies and parasite intensity in the malaria protected Fulani of West Africa. This study aimed to investigate the possible impact of *IL4*-590C/T polymorphism on anti-*P. falciparum *IgG subclasses and IgE antibodies levels and the alteration of malaria severity in complicated and uncomplicated malaria patients with or without previous malaria experiences.

**Methods:**

Anti-*P.falciparum *IgG subclasses and IgE antibodies in plasma of complicated and uncomplicated malaria patients with or without previous malaria experiences were analysed using ELISA. *IL4*-590 polymorphisms were genotyped using RFLP-PCR. Statistical analyses of the IgG subclass levels were done by Oneway ANOVA. Genotype differences were tested by Chi-squared test.

**Results:**

The *IL4*-590T allele was significantly associated with anti-*P. falciparum *IgG3 antibody levels in patients with complicated (*P *= 0.031), but not with uncomplicated malaria (*P *= 0.622). Complicated malaria patients with previous malaria experiences carrying *IL4*-590TT genotype had significantly lower levels of anti-*P. falciparum *IgG3 (*P *= 0.0156), while uncomplicated malaria patients with previous malaria experiences carrying the same genotype had significantly higher levels *(P *= 0.0206) compared to their *IL4*-590 counterparts. The different anti-*P. falciparum *IgG1 and IgG3 levels among IL4 genotypes were observed. Complicated malaria patients with previous malaria experiences tended to have lower IgG3 levels in individuals carrying TT when compared to CT genotypes (*P *= 0.075). In contrast, complicated malaria patients without previous malaria experiences carrying CC genotype had significantly higher anti-*P. falciparum *IgG1 than those carrying either CT or TT genotypes (*P *= 0.004, *P *= 0.002, respectively).

**Conclusion:**

The results suggest that *IL4*-590C or T alleles participated differently in the regulation of anti-malarial antibody isotype profiles in primary and secondary malaria infection and, therefore, could play an important role in alteration of malaria severity.

## Background

Anti-*Plasmodium falciparum *specific antibodies play a critical role in immune protection against asexual blood stages of the parasite, in which anti-*P. falciparum *IgG antibodies involved in reducing severity of the disease [1]. In particular, the cytophilic IgG1 and IgG3 subclasses are considered to protect against *P. falciparum*, whereas IgG2 and IgM are not, and even suggested to block protective effects of the former Ig subclasses [2]. Anti-*P. falciparum *IgE, as well as total IgE antibodies, which are elevated in individuals exposed to malaria in Thailand, have been implicated to play a pathogenic role during malaria infection [1]. In contrast, the anti-*P. falciparum *IgE levels in asymptomatic individuals in Tanzania were associated with a reduced risk for subsequent malaria disease [3].

The regulation of antibody profiles in patients with complicated and uncomplicated malaria is still largely unknown. In both human and mice, different cytokines are thought to induce particular Ig isotypes. In humans, IL4 regulates B cells to express the γ1, γ2, γ3, γ4, α1, α2 and ε, germline gene transcripts (GLT) and to secrete the corresponding proteins [4-6]. IL10 promotes isotype switching from IgM to IgG1, IgG3, IgG4 and/or IgE [7-9] while IFN-γ promotes IgG2 [10,11]. The inter-individual variation in cytokine production may be reflected by polymorphisms in regulatory region of the corresponding genes. The *IL4*-590 C/T transition in *IL4 *promoter was shown to influence the IL4 production as well as the elevated levels of total IgE [12,13]. The *IL4*-590T allele corresponded to *IL4*-524T and *IL4*-589T alleles in an alternative numbering scheme [14-16]. In the Fulani tribe in West Africa, *IL4*-524T allele was found to be associated with the elevated levels of anti-*P. falciparum *IgG antibodies and protection against malaria [14], while *IL4*-589T allele was associated with the elevated levels of total IgE in children with severe malaria living in Burkina Faso [15]. Conversely, total IgE levels were significantly elevated in children with cerebral malaria who carried *IL4*-590T allele and have been living in Ghana [16]. Thus, the role of *IL4*-590T allele in regulating antibody profiles and malaria severity is controversial. In mice infected with *Plasmodium chabaudi chabaudi*, the specific IgG2a and IgG3 antibodies are predominant in primary polyclonal B cell activation. At that point, IFN-γ is markedly stimulated, while IL4 is moderately enhanced. In secondary IgG1-restricted response, only IL4 is produced [17]. According to previous findings, the *IL4*-590 C/T polymorphism influences the balance between IL4 and IFN-γ and thus, could alter the severity of malaria [18]. When the same sets of sera were used subsequently to determine the anti-*P. falciparum *IgG subclasses and IgE antibodies, the results showed different regulation in patients with complicated and uncomplicated malaria [19].

Therefore, it is of interest to evaluate the impact of *IL4*-590 C/T single nucleotide polymorphism (SNP) on the production of anti-*P. falciparum *-IgG1, -IgG2, -IgG3, IgE antibodies and to determine whether such polymorphism and specific antibodies levels were associated with previous malaria experiences and clinical outcome of the disease in people living in a malaria endemic areas in Thailand.

## Methods

### Malaria subjects

The same sets of patients previously reported were studied [18,19]. Briefly, 110 and 169 patients with complicated (CM) and uncomplicated malaria (UCM), respectively, who had been living at Thai-Myanmar border in the west and Thai-Cambodia border in the east of Thailand; where malaria is endemic; were enrolled. These areas are considered as having low and seasonal malaria transmission. The annual malaria incidence rates in 2001 were 2 to 6/1,000 population [20]. The two groups of patients were matched for age (*P *= 0.849), gender (*P *= 0.137) and nationality, *i.e *Thai, Burmese, Mon and Karen (*P *= 0.614). The median age was 25 years (ranged 14-67) for complicated and 24 years (ranged 13-65) for uncomplicated malaria. Based on the interview and clinical records, 27.3% of patients with complicated and 55% of those with uncomplicated malaria had previous malaria experiences. The number of previous malaria episodes in these patients varied widely, describing mostly 1-2 episodes of malaria (84/125), 3-8 episodes (27/125) and many episodes (12/125). However, the duration of malaria infection upon admission and the previous malaria episodes among patients were far different, varying from one month to more than 10 years (mostly between one month and two years). However, both groups of patients had acute malaria infection upon admission. Therefore, complicated malaria patients with or without previous malaria experiences (CME, CMN, respectively) and uncomplicated malaria patients with or without previous malaria experiences (UCME, UCMN, respectively) were identified. Clinical manifestations of complicated and uncomplicated malaria were defined according to World Health Organization criteria [21]. Cerebral malaria was defined as unrousable coma with positive asexual forms of *P. falciparum *in blood smears, while other causes of coma were excluded. Severe malaria but not cerebral (non-cerebral severe malaria) was defined as patients exhibiting one or more of the following signs, hyperparasitaemia (>250,000 parasite/ml), hypoglycaemia (glucose <22 nmol/l), severe anaemia (haematocrit <20% or haemoglobin <7.0 g/d) or increased serum level of creatinine more than 3.0 mg/dl. The patients with complicated malaria comprised of all severe forms including cerebral malaria. Uncomplicated malaria was characterized by a positive blood smear and fever without other causes of infections and no manifestations of severe malaria as described. The patients were transported for admission to the Hospital for Tropical Diseases, Faculty of Tropical Medicine, Mahidol University, Bangkok. The study has been ethically approved by the Institute Review Board of the Faculty of Tropical Medicine, Mahidol University, Bangkok and the fully informed consent was obtained from all patients. Other details about the patients are delineated in Table 1.

**Table 1:** Characteristics of complicated and uncomplicated malaria patients

Characteristics	Complicated malaria (110)^a^	Uncomplicated malaria (169)^a^	*P*-value^b^
Age^c^(range)	25.0 (14-67)	24.0 (13-65)	0.849
			
Sex (male/female)	75/35	133/36	0.137
			
Nationality^d^			
Burmese	17 (15.5)	24 (14.2)	0.614
Karen	18 (16.4)	27 (16.0)	
Mon	37 (33.6)	72 (42.6)	
Thai	38 (34.5)	45 (26.6)	
Laos	0	1 (0.6)	
			
Blood groups^d^			
A	21 (19.1)	27 (16.0)	0.556
AB	6 (5.5)	7 (4.1)	
B	34 (30.9)	58 (34.3)	
O	47 (42.7)	66 (39.1)	
NR	2 (1.8)	11 (6.5)	
			
Haemoglobin variants*^d^			
Hb variants	14 (12.7)	29 (17.2)	0.825
Normal	95 (86.4)	133 (78.7)	
NR	1 (0.9)	7 (4.1)	
			
G6PD deficiency^d^			
Deficiency	13 (11.8)	14 (8.3)	0.597
Normal	96 (87.3)	153 (90.5)	
NR	1 (0.9)	2 (1.2)	
			
Parasitaemia^e ^(range)	117 322 (33-1 336 900)	5641 (17-238 800)	<0.0001
			
Highest fever°C^f^	38.1 ± 1.005	38.4 ± 0.981	0.011
			
Haemoglobin g/d^f^	11.4 ± 0.24	12.1 ± 0.17	0.021
			
Creatinine mg/dl^f^	1.36 ± 1.21	0.88 ± 0.37	<0.0001
			
Glucose nmol/l^f^	131.47 ± 62.06	118.17 ± 28.78	0.016
			
Splenomegaly^g^			
Positive	18 (16.4)	3 (1.8)	0.039
Negative	92 (83.4)	166 (98.2)	
			
Hepatomegaly^g^			
Positive	66 (60)	32 (18.9)	< 0.0001
Negative	44 (40)	137 (81.1)	
			
Previous malaria episodes^d^			
Yes	30 (27.3)	93 (55.0)	< 0.0004
No	78 (70.9)	76 (45)	
NR	2 (1.8)	0	

a = Number of patients, b = Mann Whitney *U *or unpaired *t *test used for the analysis, c = Median, d = Percentages (in the parenthesis), e = Geometric mean/ml of blood, f = Mean ± SD, g = Percentages of individual with enlarged spleens and/or liver are in the parenthesis. *The Hemoglobin (Hb) variants includes HbE, HbF. NR = No report.

### Blood samples

Blood samples from patients with complicated and uncomplicated malaria were collected in EDTA sterile tubes before treatment on admission. The plasma and packed cells were separated and frozen at -20°C until use.

### Microscopic examination

Thick and thin films were made for microscopic examination after standard Giemsa staining. Parasite densities were determined by calculating *P. falciparum *parasites per ml of blood obtained from the number of parasites observed per 1,000 erythrocytes in thin film or per 200 leucocytes in thick film.

### Malaria antigens

The laboratory *P. falciparum *strain F32 was used to prepare the antigens for malaria antibodies detection. The late stages of *P. falciparum *infected erythrocytes were enriched by 60% Percoll and sonicated as described [22].

### Detection of anti-*P. falciparum *IgG subclasses and IgE antibody levels

Anti-*P. falciparum *-IgG1, -IgG2, -IgG3 and IgE antibodies levels were determined by enzyme-linked immunosorbent assay (ELISA) as described previously [23] with some modification. The monoclonal antibodies used in the ELISA were given [19]. Negative control sera were from Swedish donors who have never been exposed to malaria.

### Genotyping

DNA was extracted from buffy coats by phenol-chloroform extraction [24]. Genomic DNA was used as a template for amplification by PCR and *IL4*-590 C/T polymorphism was detected by restriction fragment length polymorphism analysis as previously described [18].

### Statistical analyses

The data were analysed using SPSS or StatView computer software. The differences in characteristics of the patients with complicated and uncomplicated malaria were analyzed by unpaired *t*-test or Mann Whitney *U *test. The highest fever, haemoglobin, creatinine, and glucose levels between the two patient groups were tested by unpaired *t*-test, while the other characteristics were tested by Mann Whitney *U *test. The Chi-squared test was used to compare allele and genotype frequencies between the two patients groups. One-way ANOVA and unpaired *t*-test after log transformation were used to evaluate the significant associations between anti-*P. falciparum *IgG subclasses or IgE antibodies and the *IL4*-590 genotypes. The IgG subclasses and IgE were taken in the extreme 3 tiles. The genetic distribution of the *IL4*-590 genotypes between the low and high quartile was compared using Chi-squared test. A *P*-value of less than 0.05 was considered to be significant.

## Results

### *IL4*-590 C/T allele frequency in complicated and uncomplicated malaria patients with or without previous malaria experiences

As described previously [19], the frequency of *IL4*-590 T allele in patients with complicated malaria did not differ from those with uncomplicated malaria (0.72 in both groups). When the allele and genotype frequencies in patients with or without previous malaria experiences were explored, the allele and genotype frequencies between complicated and uncomplicated malaria groups either with or without previous malaria experiences did not differed (*P*-value allele: 0.7501 *vs *0.8755 and genotype: 0.9137 *vs *0.9459 for patients with or without previous malaria experiences, respectively) (Table 2)

**Table 2:** Allele and genotype frequencies of *IL4*-590 polymorphism in complicated and uncomplicated malaria patients with or without previous malaria experiences.

***IL4*-590 C/T**	**Genotype n (%)**			**Allele %**	
		
	**CC**	**CT**	**CC**	**C**	**T**
	
CME	2 (7.4)	11 (40.7)	14 (51.9)	27.78	72.22
UCME	7 (7.7)	33 (36.2)	51 (56.0)	25.82	74.18
*P*-value^#^		0.9137		0.7501	
	
CMN	4 (5.3)	34 (44.7)	38 (50.0)	27.63	72.37
UCMN	4 (5.4)	35 (47.3)	35 (47.3)	29.05	70.95
*P*-value		0.9459		0.8755	

^#^Chi- squared test was used for the analysis

### Association between *IL4*-590 polymorphism and anti-*P. falciparum *IgG subclasses and IgE antibody levels

The anti -*P. falciparum *-IgG1, -IgG2, -IgG3 and IgE antibody concentrations in relation to *IL4*-590 genotypes in complicated malaria (CM) and uncomplicated malaria (UCM) patients are illustrated in Table 3. In complicated malaria, the levels of anti-*P. falciparum *IgG3 were significantly different among individuals carrying *IL4*-590 TT, CT and CC genotypes (*P *= 0.031), while the levels of anti-*P. falciparum *IgG1 tended to be significant among *IL4*-590 genotype (*P *= 0.095). To study the impact between *IL4*-590 genotypes on anti-*P. falciparum *IgG1 and IgG3 levels, the antibody levels in individuals carrying different genotypes were compared by TT to CT, TT to CC and CT to CC genotypes. As shown in Table 4, the anti-*P. falciparum *IgG1 levels were significantly different between individuals carrying TT genotype compared to those carrying CC genotype (median 18.04 *vs *223.80: *P *= 0.025) and between individuals carrying CT genotype compared to those carrying CC genotype (median 24.12 *vs *223.80, *P *= 0.037). In contrast, the anti-*P.falciparum *IgG3 levels were significantly different only between individuals carrying TT compared to those carrying CT genotype (median 0.51 *vs *1.20, *P *= 0.030). When complicated malaria patients with or without previous malaria experiences (CME and CMN, respectively) were analysed separately, similar patterns were seen by the anti-*P. falciparum *IgG1 levels were significantly different between individuals carrying TT and CC genotypes (median 8.54 *vs *223.80: *P *= 0.002) as well as between CT and CC genotypes (median 13.94 *vs *223.80: *P *= 0.004). Interestingly, these observations were seen only in complicated malaria patients without previous malaria experiences. While the difference in anti-*P. falciparum *IgG3 levels between patients carrying TT and CT genotype tended to be significant in complicated malaria patients with previous malaria experiences (median 1.32 *vs *5.62: *P *= 0.075). No such tendency could be observed in the levels of anti-*P. falciparum *IgG2 and IgE in complicated malaria patients either with or without previous malaria experiences. Among uncomplicated malaria patients carrying TT, CT or CC genotypes, the levels of anti-*P. falciparum*-IgG1, -IgG2, -IgG3 and IgE did not differ.

**Table 3:** Anti-*P. falciparum *IgG subclass and IgE levels (median) separated by *IL4*-590 CT genotype in patients with complicated and uncomplicated malaria (CM, UCM).

		CC		CT		TT	
	
	N	Median	N	Median	N	Median	*P*-value^#^
**CM**							
Anti-*Pf*IgG1*	5	223.80	46	24.12	53	18.04	**0.096**
Anti-*Pf*IgG2*	5	6.05	46	2.03	53	2.79	0.112
Anti-*Pf*IgG3*	5	1.51	46	1.20	53	1.52	**0.031**
Anti-*Pf*IgE**	5	0.43	47	0.62	53	2.36	0.915

**UCM**							
Anti-*Pf*IgG1*	11	252.80	67	39.32	84	88.21	0.195
Anti-*Pf*IgG2*	11	7.60	68	4.40	83	5.77	0.299
Anti-*Pf*IgG3*	11	2.65	68	2.69	85	3.13	0.622
Anti-*Pf*IgE**	11	0.96	68	0.65	85	0.76	0.892

* = μg/ml, ** = ng/ml, N = number of patients, ^#^Statistical analysis of differences

in IgG subclass levels among the different genotypes tested with ANOVA.

**Table 4:** Anti-*P. falciparum *IgG1 and IgG3 levels (median) according to *IL4*-590 genotypes in complicated malaria patients with or without previous malaria experiences.

		CM		
	
	CC (5)^a^	CT (46)^a^	TT (53)^a^	*P*-value^#^
Anti-*Pf*IgG1*	-	24.12	18.04	0.628
	**223.80**	-	**18.04**	**0.025**
	**223.80**	**24.12**	-	**0.037**

Anti-*Pf*IgG3*	**-**	**1.20**	**0.51**	**0.030**
	1.51	**-**	0.51	0.159
	1.51	1.20	**-**	0.455

		**CME**		
	
	CC (2)	CT (11)	TT (14)	*P*-value

Anti-*Pf*IgG1*	**-**	31.13	53.04	0.983
	280.43	-		0.835
	280.43	31.13	53.04	0.830

Anti-*Pf*IgG3*	**-**	**5.62**	**1.32**	**0.075**
	8.06	**-**	1.32	0.384
	8.06	5.62	**-**	0.961

		**CMN**		
	
	CC (3)	CT (33)	TT (38)	*P*-value

Anti-*Pf*IgG1*	**-**	13.94	8.54	0.636
	**223.80**	-	**8.54**	**0.002**
	**223.80**	**13.94**	-	**0.004**

Anti-*Pf*IgG3*	**-**	0.80	0.40	0.217
	1.51	-	0.40	0.154
	1.51	0.80	-	0.274

* = μg/ml, ^#^Statistical analysis of differences in IgG subclass levels

between the different genotypes tested with unpaired *t*-test,

a = Number of patients (in the parenthesis).

To investigate whether the anti-*P. falciparum *IgG subclasses and IgE were associated with certain *IL4*-590 genotypes and whether the distribution of the C and/or T allele differed between patients with complicated and uncomplicated malaria either with low or high specific antibody levels, the antibody profiles were divided into three tiles, *i.e*. those with low, medium and high antibody levels. This division was based on the findings that there was a great individual variation in antibody responses to the crude plasmodium antigens. Because of the low numbers of homozygotes for the *IL4*-590 C allele frequency, the individuals homozygous and heterozygous for C alleles (CC+CT) were pooled and compared with those homozygous for T alleles. In complicated malaria, individuals homozygous for T alleles had significantly lower anti-*P. falciparum *IgG3 antibody (*P *= 0.0054), while no significant difference was found in uncomplicated malaria (*P *= 0.079). Conversely, the *IL4*-590 genotypes were not significantly associated either with anti-*P. falciparum *-IgG1, -IgG2 or with anti-*P. falciparum *IgE antibodies when low and high tiles in complicated and uncomplicated malaria were compared (Table 5). When the results were analysed separately according to the history of individual's malaria experiences, only individuals homozygous for T alleles had significantly lower anti-*P. falciparum *IgG3 antibody in complicated malaria patients with previous malaria experiences (CME) (*P *= 0.0156) while higher anti-*P. falciparum *IgG3 antibody levels were seen in uncomplicated malaria patients with previous malaria experiences (UCME) (*P *= 0.0206). In contrast, no such associations could be found when the *IL4*-590 genotypes were compared between complicated malaria patients without previous malaria experiences (CMN) and uncomplicated malaria patients. Conversely, high anti-*P. falciparum *IgG1 antibody levels seem to be associated with the *IL4*-590TT genotype in uncomplicated malaria patients with previous malaria experiences (UCME) (*P *= 0.1219) (Table 6). No significant associations between *IL4*-590 genotypes and anti-*P. falciparum *IgG2 and IgE antibodies were found when the low and the high tiles were compared in all CME, CMN, UCME and UCMN (uncomplicated malaria patients without previous malaria experiences)

**Table 5:** Association between *IL4*-590 genotype and anti-*P. falciparum *-IgG1, -IgG2, -IgG3 and IgE low or high tiled levels in patients with complicated and uncomplicated malaria.

				**Complicated**				**Uncomplicated**			
					
	**3-tiles of Ig levels**		**N**	**CC**	**CT**	**TT**	***P*-value^#^**	**CC**	**CT**	**TT**	***P*-value^#^**
	
Anti-*Pf *IgG1			274	5^A^	46^B^	53^C^		11^A^	67^B^	84^C^	
Anti-*Pf *IgG2			274	5	46	53		11	68	83	
Anti-*Pf *IgG3			276	5	46	53		11	68	85	
Anti-*Pf *IgE			277	5	47	53		11	68	85	
	
Anti-*Pf*IgG1*	Low	0-14.47	91^D^	0	20	26	0.395	2	23	16	0.118
	Medium	14.76-106.1	92	2	16	16		3	20	32	
	High	107.2-1874	91	3	10	11		6	24	36	
	
Anti-*Pf*IgG2*	Low	0.414-2.384	91	0	26	23	0.856	2	23	17	0.179
	Medium	2.386-6.617	92	3	12	22		3	19	29	
	High	6.629-177	91	2	8	8		6	26	37	
	
Anti-*Pf*IgG3*	Low	0.053-0.798	92	**1**	**19**	**30**	**0.0054**	**3**	**20**	**16**	**0.079**
	Medium	0.854-3.55	92	2	13	18		5	22	28	
	High	3.629-119	92	**2**	**14**	**5**		**3**	**26**	**41**	
	
Anti-*Pf *IgE**	Low	0.015-0.3851	92	2	15	17	0.896	3	28	25	0.564
	Medium	0.3854-1.332	93	2	17	21		4	14	30	
	High	1.346-29.05	92	1	15	15		4	26	30	

N = Number of plasma for determining IgG1, IgG2, IgG3, IgE and genotyping *IL4*-590, # = Chi-squared test was used to test the difference of *IL4*-590 CC+CT and TT distribution between the low and high 3 tiles. * = μg/ml, ** = ng/ml, A = number of patients who carry *IL4*-590 CC genotype, B = number of patients who carry *IL4*-590 CT genotype, C = number of patients who carry *IL4*-590 TT genotype, D = Number of patients who had low, middle and high anti-*P. falciparum *-IgG1, -IgG2, -IgG3 and IgE levels.

**Table 6:** Association between *IL4*-590 genotype and anti-*P. falciparum *-IgG1 and -IgG3 levels base on previous experiences malaria infection in patients with complicated and uncomplicated malaria.

			Complicated malaria							
			
			Without previous malaria experiences (76)^a^				With previous malaria experiences (27)			
				
	3-tiles of Ig levels		CC	CT	TT	*P*-value^#^	CC	CT	TT	*P*-value^#^
	
Anti-*Pf*IgG1*	Low	0-14.47	0^A^	17^B^	21^C^	0.1373	0	3	4	0.7715
	Medium	14.76-106.1	1	11	11		1	4	5	
	High	107.2-1874	2	5	6		1	4	5	
	
Anti-*Pf*IgG3*	Low	0.053-0.798	0	17	23	0.2136	1	2	6	0.0156
	Medium	0.854-3.55	2	10	11		0	2	7	
	High	3.629-119	1	6	4		1	7	1	
			**Uncomplicated malaria**							
			
			**Without previous malaria experiences (74)**				**With previous malaria experiences (91)**			
				
			**CC**	**CT**	**TT**	***P*-value^#^**	**CC**	**CT**	**TT**	***P*-value^#^**
	
Anti-*Pf*IgG1	Low	0-14.47	0	15	11	0.8019	**2**	**7**	**5**	**0.1219**
	Medium	14.76-106.1	2	9	12		1	11	20	
	High	107.2-1874	2	11	11		**4**	**13**	**25**	
	
Anti-*Pf*IgG3	Low	0.053-0.798	1	13	12	0.8772	**2**	**7**	**4**	**0.0206**
	Medium	0.854-3.55	1	10	12		4	11	16	
	High	3.629-119	2	12	11		**1**	**14**	**30**	

a = Number of patients (in the parenthesis), # = Chi- squared test was used to test the difference of *IL4*-590 CC, CT and TT distribution between the low and high 3 tiles, * = μg/ml, A, B and C = number of patients with previous malaria experiences for determining anti-*P. falciparum *-IgG1, -IgG3 and carry *IL4*-590 CC, CT, TT genotypes, respectively.

## Discussion

This study demonstrated that *IL4*-590 genotypes could differently regulate anti-*P. falciparum *-IgG1 and -IgG3 responses. The association between individuals homozygous for *IL4*-590 T alleles and the levels of anti-*P. falciparum *IgG3 was found only in patients who had previous malaria experiences with low levels in complicated malaria and high levels in uncomplicated malaria. When the differences in antibody levels between genotypes were explored, similar results were seen for anti-*P. falciparum *IgG3 in that complicated malaria patients with previous malaria experiences tended to have lower IgG3 levels in those carrying TT compared to CT genotypes. In contrast, the complicated malaria patients without previous malaria experiences who carried CC genotype had significantly higher IgG1 than those carrying either CT or TT genotypes. Thus, repeated exposure and host genetics, *i.e *the polymorphism of *IL4*-590 or other related genes has an impact on the modulation of antibody isotype profiles and altered clinical outcome.

The relationship between *IL4 *polymorphism and anti-*P. falciparum *IgG subclasses antibody levels has not been studied thoroughly. This is the first finding to show that a single nucleotide polymorphism at position *IL4*-590 transition from C to T is associated with the levels of anti-*P. falciparum *IgG3 antibody in malaria patients. In complicated malaria especially those with previous malaria experiences, the levels of anti-*P. falciparum *IgG3 were significantly different among patients carrying *IL4*-590 CC, CT or TT genotypes. In particular, complicated malaria patients homozygous for *IL4*-590 T alleles and with previous malaria experiences exhibited significantly lower levels of anti-*P. falciparum *IgG3 antibody than their *IL4*-590 counterparts, whereas significantly higher levels were found in uncomplicated malaria patients homozygous for *IL4*-590 T alleles and with previous malaria experiences. Similar trends were observed for the anti-*P. falciparum *IgG1 in uncomplicated malaria patients with previous malaria experiences although this did not reach statistically significant differences. These results are consistent with the previous findings that low levels of IgG3 may lead to the onset of complicated malaria while high IgG3 antibody levels may protect against malaria [25,26] and with the demonstration on the induction of CD40L stimulated naïve B cells by IL4 alone or in combination with IL10 to express IgG1, IgG2 and IgG3, but little IgG4 [27]. IgG3 can activate the complement pathways [28] and co-operate through FcR receptors [29], thus, IgG3 could induce cell mediated lysis via complement pathways, phagocytosis and/or ADCI mechanism [2,30] which are all known to facilitate parasite clearance. In fact, IgG3 may involve in anti-disease immunity by neutralizing parasite toxin, the glycosylphosphatidylinositol (GPI) membrane anchors of *P. falciparum *surface proteins and/or inhibiting cytoadherence of infected red blood cells, thus, interfering the parasite sequestration [31,32]. In humans, IL10, IL21 are associated with IgG1 and IgG3 antibody production [7,8,33] while IL-27 is modest and regulates exclusively the production of IgG1 [34]. Furthermore, the interaction and combination of different base changes at several sites in the promoter and/or the co-localized alleles of these cytokines and their signaling genes including the *IL4*-590 polymorphism may differ in patients with complicated and uncomplicated malaria and thus, alter the IgG1 and IgG3 production. This may also explain why different anti-*P. falciparum *IgG3 levels were observed only among patients with complicated malaria carrying *IL4*-590 TT genotype compared to their counterparts.

The present study did not reveal any association between *IL4-*590 C/T transition and the severity of malaria in either with or without previous malaria experiences, although there was an association between *IL4-*590 TT genotype and the levels of anti-*P. falciparum *IgG3 antibody in both complicated and uncomplicated malaria patients with previous malaria experiences. The reason for this discrepancy is probably due to: firstly, the polymorphism itself may not be functional but act as genetic markers of other functional polymorphisms; secondly, the polymorphisms may directly regulate IL4 production only in the presence of other genetic determinants. In contrast to the results recently demonstrated in Mali of West Africa. The *IL4-*590 T alleles were associated with the increased levels of both total and anti-malarial IgE in the Fulani, but not the Dogon. However, *IL4-*590 polymorphism did not influence on the levels of anti-malarial IgG1-3 subclasses in both the Fulani and the Dogon [35]. The reason for genetic disparities can be attributed to different ages, ethnicity, geographical differences, and clinical feature as asymptomatic and acute malaria infection.

The *IL4*-590 C or T alleles in association with primary and secondary anti-*P. falciparum -*IgG1 and -IgG3 responses were different. The *IL4*-590 T allele was associated with anti-*P. falciparum *IgG3 levels in which the low levels were observed in complicated and the high levels were observed in uncomplicated malaria with previous malaria experiences. In complicated malaria with previous malaria experiences, the anti-*P. falciparum *IgG3 levels tended to be lower in patients carrying TT than those carrying CT genotypes. Conversely, the *IL4*-590 T allele seems to be associated with high anti-*P. falciparum *IgG1 in only uncomplicated malaria patients with previous malaria experiences, whereas, complicated malaria patients without previous malaria experiences who carried CC genotype had significantly higher anti-*P. falciparum *IgG1 than those either with CT or TT genotypes. Therefore, it is likely that T allele associated with secondary malaria infection, while C allele associated with primary infection. The shift of Ig-isotypes from primary to secondary responses has been demonstrated in mouse models to be correlated with the levels of either Th1 or Th2 cytokines [17]. Th1 subset predominates in the non-specific primary response, whereas Th2 is the major activated pathway in the secondary specific response. As demonstrate earlier that the nuclear factor of activated T cells (NFAT) binding site at *IL4*-590 T creates a hair trigger for *IL4 *transcription, it is advantageous when a Th2 response is required, while *IL4*-590 C is favored when a Th1 response is required [36]. Therefore, the modulation of Th1 and Th2 cytokines productions could have the impact on antibody classes and subclasses productions. It is possible that *IL4*-590°C or T in combination with previous exposure to malaria antigens probably partly modify the antibody isotype profiles and thus represent a wide spectrum of clinical symptoms. However, some of these observations need to be confirmed in a larger sample size in order to correct the confounding factors, such as age, number of previous episodes and parasite-virulence.

## Conclusion

This study showed that *IL4*-590 C or T is likely to play a modulating role in the regulation of antibody profiles in primary and secondary immune responses. The *IL4*-590 T allele participated in the regulation of anti-*P. falciparum *-IgG1 and -IgG3 in secondary infection, whereas *IL4*-590 C allele might participated in the regulation of anti-*P. falciparum *IgG1 in primary infection. In individuals carrying the *IL4*-590 TT genotype with previous malaria experiences, an increased risk of complicated malaria was associated with low anti-*P. falciparum *IgG3 antibody, whereas a lower risk was associated with high antibody levels. However, a panel of genetic variations in humoral immune responses and their related genes in larger sample size should be further investigated which may provide clue for better understanding in the alteration of disease severity.

## List of abbreviations used

CM: complicated malaria patients; CME: complicated malaria patients with previous malaria experiences; CMN: complicated malaria patients without previous malaria experiences; UCM: uncomplicated malaria patients; UCME: uncomplicated malaria patients with previous malaria experiences; UCMN: uncomplicated malaria patients without previous malaria experiences.

## Competing interests

The authors declare that they have no competing interests.

## Authors' contributions

SK and PT designed the study; PT prepared the blood samples, performed the *IL4*-590 genotyping and ELISA, collected clinical data, analysed statistics and drafted the manuscript; HP contributed to ELISA; MH assisted in *IL4*-590 genotyping; SK and MTB supervised the work, revised the manuscript and financed the research. All authors read and approved the final manuscript.
